# Novel artificial intelligence algorithms for diabetic retinopathy and diabetic macular edema

**DOI:** 10.1186/s40662-024-00389-y

**Published:** 2024-06-17

**Authors:** Jie Yao, Joshua Lim, Gilbert Yong San Lim, Jasmine Chiat Ling Ong, Yuhe Ke, Ting Fang Tan, Tien-En Tan, Stela Vujosevic, Daniel Shu Wei Ting

**Affiliations:** 1 Singapore National Eye Centre, Singapore Eye Research Institute, 11 Third Hospital Avenue, Singapore, 168751 Singapore; 2 Duke-NUS Medical School, Singapore, Singapore; 3 SingHealth AI Health Program, Singapore, Singapore; 4 Division of Pharmacy, Singapore General Hospital, Singapore, Singapore; 5 Department of Anesthesiology and Perioperative Science, Singapore General Hospital, Singapore, Singapore; 6 Department of Biomedical, Surgical and Dental Sciences, University of Milan, Milan, Italy; 7 Eye Clinic, IRCCS MultiMedica, Milan, Italy

**Keywords:** Artificial intelligence, Deep learning, Diabetic retinopathy, Retinal imaging, Telemedicine

## Abstract

**Background:**

Diabetic retinopathy (DR) and diabetic macular edema (DME) are major causes of visual impairment that challenge global vision health. New strategies are needed to tackle these growing global health problems, and the integration of artificial intelligence (AI) into ophthalmology has the potential to revolutionize DR and DME management to meet these challenges.

**Main text:**

This review discusses the latest AI-driven methodologies in the context of DR and DME in terms of disease identification, patient-specific disease profiling, and short-term and long-term management. This includes current screening and diagnostic systems and their real-world implementation, lesion detection and analysis, disease progression prediction, and treatment response models. It also highlights the technical advancements that have been made in these areas. Despite these advancements, there are obstacles to the widespread adoption of these technologies in clinical settings, including regulatory and privacy concerns, the need for extensive validation, and integration with existing healthcare systems. We also explore the disparity between the potential of AI models and their actual effectiveness in real-world applications.

**Conclusion:**

AI has the potential to revolutionize the management of DR and DME, offering more efficient and precise tools for healthcare professionals. However, overcoming challenges in deployment, regulatory compliance, and patient privacy is essential for these technologies to realize their full potential. Future research should aim to bridge the gap between technological innovation and clinical application, ensuring AI tools integrate seamlessly into healthcare workflows to enhance patient outcomes.

## Background

Diabetes mellitus (DM) and its major ocular complications of diabetic retinopathy (DR) and diabetic macular edema (DME) are becoming global health challenges of significant magnitude. Estimates by the International Diabetes Federation project a rise in cases of diabetes over the next 20 years toward a staggering 700 million by the year 2045 [1]. Paralleling this rise in systemic disease, a recent systematic review and meta-analysis also estimated increases in the global burden of DR and DME to 160.5 million and 28.61 million cases, respectively, by 2045 [2]. This dramatic rise in caseload is expected to pose a significant strain on healthcare resources, emphasizing the need for advanced solutions to effectively manage and address these challenges in the coming years. The integration of artificial intelligence (AI) into the field of ophthalmology, particularly in the management of DR and DME, marks a significant paradigm shift towards improving diagnostic and therapeutic outcomes for these diabetes-associated ocular diseases [3]. AI, encompassing machine learning (ML) and its more advanced subset, deep learning (DL), employs algorithms and neural networks to enable systems to learn from data and analyze complex patterns. This progression from ML to DL is yielding increasingly effective models, significantly improving the field's diagnostic and analysis capabilities.

**Fig. 1:**
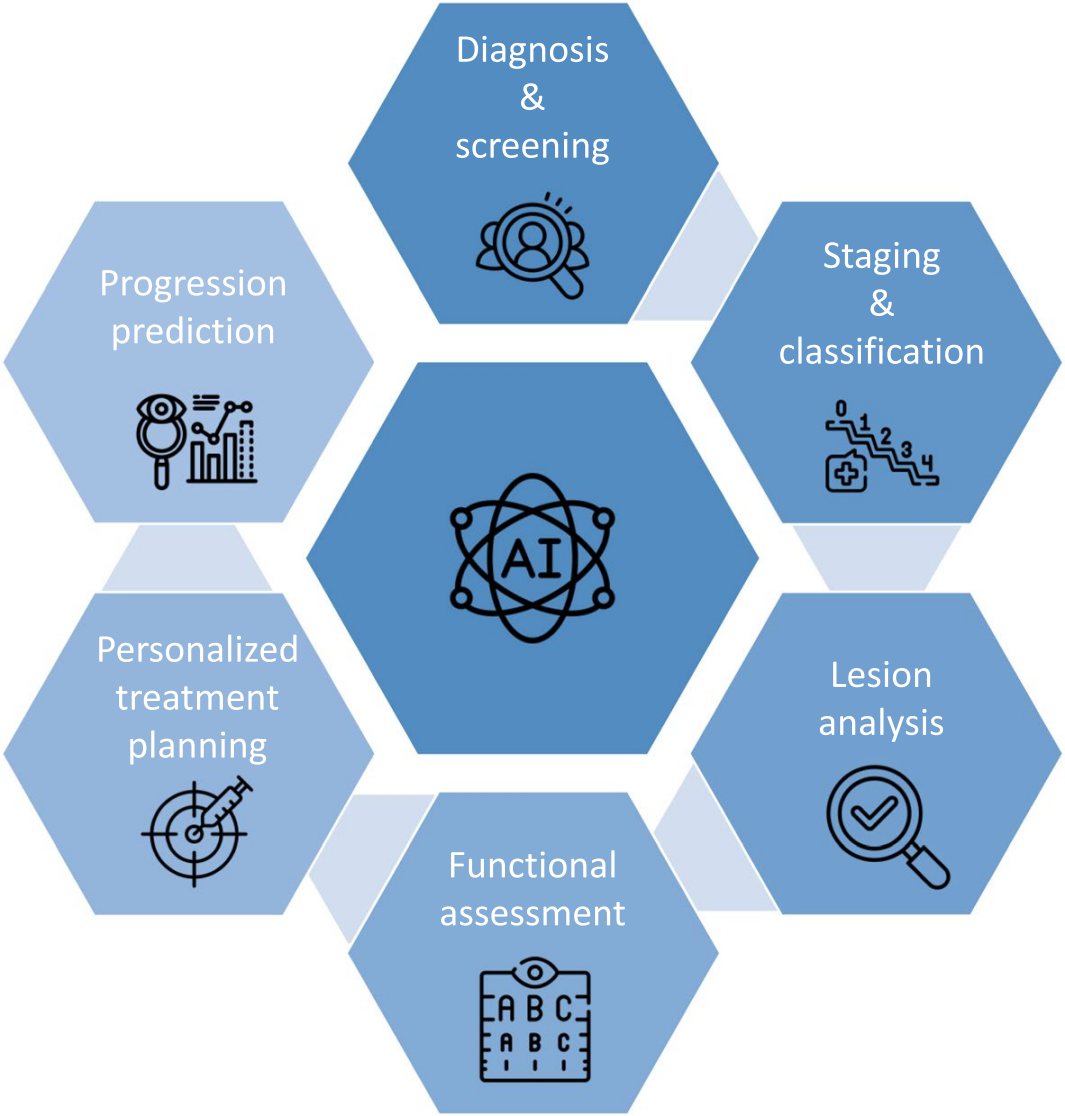
Overview of current artificial intelligence models for various applications in diabetic retinopathy and diabetic macular edema

In the span of the last decade, the rise of AI in healthcare has not only brought the potential tools to address the significant rise in DR and DME caseload, but also radically impact the ways in which DR and DME can be diagnosed and subsequently managed and monitored. AI-based DR screening systems have emerged as valuable tools for reducing screening workloads, with numerous algorithms now commercially available or in clinical use. Additionally, AI algorithms are advancing in areas such as lesion analysis, disease progression prediction, and personalized management, offering promising results (Fig. 1). However, despite the significant advancements in AI algorithms for diagnosing and managing DR and DME, challenges related to real-world effectiveness, regulatory compliance, and privacy concerns persist.

The objective of this review is to provide a comprehensive overview of the latest AI algorithms for DR and DME, discuss their advancements and limitations, and the technical advancement that can address the challenge of development and deployment in real-world settings, assess the challenges in real-world deployment, and outline future directions for research and clinical implementation. Through this comprehensive analysis, we aim to contribute to the ongoing advancements in AI-driven ophthalmic care, ultimately improving outcomes for individuals with diabetes.

## Main text

### Methodology

To assess the current landscape of AI models related to DR and DME, we conducted a comprehensive literature review through Google Scholar and PubMed, considering studies published up to August 5, 2023. Our search strategy incorporated a range of keywords, including "diabetic retinopathy", "diabetic macular edema", "fundus photograph", "optical coherence tomography", "artificial intelligence", "machine learning", and "deep learning". We limited our selection to articles published in English. When encountering multiple publications from the same study, we considered them as a single entry. Exclusions were made for articles that were published before 2019 or did not present original research utilizing ML or DL techniques or not for DR or DME.

### AI in screening and diagnosis of DR

With the growing diabetic population worldwide, the demand for more efficient DR screening methods is increasing, highlighted by their cost-effectiveness and the widespread recommendation for regular screening. Numerous studies on DR screening models using fundus photos have led to the transition of some from experimental development to clinical practice over the years, marking a significant advancement in ophthalmology [4, 5]. These AI systems have progressed from solely detecting DR to identifying other eye diseases like age-related macular degeneration and glaucoma. Primarily aimed at detecting referable diabetic retinopathy (RDR) with high accuracy, AI-driven models provide scalable, efficient, and precise screening solutions. This technological evolution significantly surpasses traditional methods and human graders in efficiency, ushering in an era of automated, large-scale DR screening programs globally.

While these AI systems have shown promise in clinical trials and received regulatory approvals, their performance in real-world settings has yet to be fully convincing. Real-world evaluations of several state-of-the-art AI DR screening systems have revealed inconsistent performance, with sensitivities ranging from 50.98% to 85.90%, and specificities from 60.42% to 83.69% [6]. Similarly, a real-world test of Google's AI system in Thailand revealed a lower specificity compared to the human graders [7]. A high false negative rate risks overlooking diseased individuals, while a high false positive rate may result in unnecessary referrals, raising significant concern regarding the cost-effectiveness of these AI implementations. This performance gap may stem from various factors such as limited diversity and representativeness in the training datasets regarding patient demographics, image acquisition methods, variations in image quality outside controlled environments, and potential overfitting to specific data characteristics. It emphasizes the importance of continuously refining and adapting AI algorithms to enhance their applicability and effectiveness across different populations and clinical settings.

As the field of ophthalmology continues to evolve, the transition from traditional imaging techniques to more advanced imaging modalities has been evident. Ultra-widefield (UWF) imaging captures a greater extent of the peripheral retina, which may provide more prognostic information and allow for more accurate DR diagnosis and grading. In 2021, Tang et al. trained a DL system using 2,861 UWF fundus photos, aiming to assess image gradability and detect both vision-threatening DR and RDR. This model demonstrated a high level of diagnostic performance, consistently surpassing an area under the receiver operating characteristic curve (AUC) of 0.90 across external validation datasets from three countries [8]. Similarly, IDx-DR (IDx Technologies, USA), which was primarily designed for traditional fundus photos, was found to surpass human graders in identifying asymptomatic RDR on UWF images [9]. Despite their relative expense and limited availability making them less convenient tools, UWF images hold potential for further AI research in comprehensive DR assessment.

Another innovative approach involves integrating multiple imaging modalities. While single-modality analysis offers valuable insights, it often provides a limited perspective of complex conditions. Multi-modal analysis, on the other hand, integrates data from various sources, offering a more comprehensive and potentially more nuanced understanding. Hua et al. proposed an architecture to coordinate hybrid information from fundus photos and widefield optical coherence tomography angiography (OCTA). The model achieved robust performance on both domestic and public datasets, with a quadratic weighted kappa rate of 0.902 on the small-sized internal dataset in DR grading, and an AUC of 0.994 in the Messidor dataset in detecting RDR [10]. Nagasawa et al. evaluated the precision of DR staging using a deep convolutional neural network (CNN) with 491 pairs of UWF fundus photos and OCTA images as input. While their results indicated effective DR detection using the combined inputs, they observed that this multi-modal approach did not significantly outperform the single-modality model in terms of diagnostic accuracy [11]. This observation suggests that multi-modal AI techniques may not always offer significantly more information across all applications. Further research is needed to assess their real-world utility and to establish optimal implementation strategies.

#### AI models for DR lesion segmentation

While DR screening AI systems provide an overall diagnostic assessment of DR, there is also a growing need for a more in-depth, granular understanding of the specific anomalies. Different AI approaches have been deployed for DR lesion segmentation in the recent 20 years, from image processing to traditional ML and DL [12]. Most of the recent segmentation models are based on CNN which can improve generalization, automatically extract features, have higher robustness to variation of image quality, and are more efficient and capable of multitasking compared to traditional ML. In recent years, generative adversarial networks (GANs) have also been used in segmentation tasks for retinal lesions [13, 14]. GANs possess the unique capability to generate synthetic images that mimic normal fundus photos. By comparing these generated normal images with diseased ones, GAN-based models can effectively identify and differentiate lesions [13].

Visualization and annotation of these lesions can be useful in clinical adoption. DR lesion segmentation systems facilitate meticulous analysis by elucidating the precise location, morphology, and extent of each lesion, and can help to quantify lesion counts in an efficient and automated manner. For example, it has been shown that quantitative analysis of DR lesions can help to better predict the risk of progression to proliferative DR (PDR) [15]. Such precision paves the way for better disease staging, personalized treatment, and objective progression tracking.

Integration of lesion detection into DR screening systems was observed to enhance diagnostic performance [16]. Dai et al. introduced a ResNet-based lesion-aware sub-network, outperforming architectures like VGG and Inception, and employed transfer learning from a pre-trained DR base network. As part of a DR screening system, DeepDR, this architecture achieved AUCs of 0.901–0.967 for lesions detection, including microaneurysms, hard exudates, cotton-wool spots and hemorrhage, and the overall DR grading achieved an average AUC of 0.955 [17]. Anderson et al. achieved improved performance by incorporating a segmentation model into a DR classification framework, with manually segmenting 34,075 DR lesions to develop a segmentation model, and then constructing a 5-step classification model [18]. Together with DR screening systems, these AI models can collectively deliver a more detailed evaluation, with screening systems acting as a broad initial assessment and lesion segmentation models providing more in-depth analysis of disease status.

#### AI models for prediction of DR progression

Beyond disease detection and classification, newer AI algorithms have been developed to predict the development and progression of DR. Individual risk factors such as patient demographics and clinical variables are well known to influence the risk of DR progression [19–21]. Structural and functional changes in ocular investigative modalities have also shown association with DR progression, such as wider retinal arteriolar caliber and localized delays on multifocal electroretinogram that corresponded to specific retinal locations where DR structural changes developed [22–24].

Al-Sari et al. used ML prediction models to predict the development of DR within five years with an AUC of 0.75 using clinical variables (e.g., albuminuria, glomerular filtration rate, DR status) and an AUC of 0.79 using blood-derived molecular data (e.g., fatty acids, amino acids, sphingolipids) [25]. Several DL algorithms based on fundus photos have also been developed to predict DR progression. Arcadu et al. predicted a 2-step worsening on the Early Treatment of Diabetic Retinopathy Study (ETDRS) scale within one year with an AUC of 0.79 [26]. Bora et al. achieved similar performance in predicting the risk of incident DR within two years. Furthermore, by incorporating clinical variables including diabetes duration and control in addition to fundus photos alone, this further enhanced the predictive performance [27]. More recently, Rom et al. developed a DL model that outperformed prior fundus photos-based algorithms in predicting risk of incident DR up to 3–5 years (AUC = 0.82), and RDR within two years (AUC = 0.81) [28]. Moreover, heatmap analyses using explainable AI techniques revealed that high attention areas of DL algorithms corresponded to regions on baseline fundus photos that eventually developed DR structural changes during follow-up visits. These indicate the potential that DL algorithms possess in uncovering subtle associations in feature-rich fundus photos in the prediction of DR progression.

Such AI models can potentially have a significant clinical impact in personalizing DR screening intervals. For example, patients with DM identified as low-risk for progression may be screened less frequently beyond the existing 1–2 yearly intervals, thereby freeing up much-needed resource capacity for patients at higher risk of disease progression, and who may need more intensive, shorter surveillance intervals. This is particularly important in the face of increasing prevalence of diabetes in increasingly aged populations. For instance, the RetinaRisk algorithm (RetinaRisk, Iceland) generates a recommended screening interval based on the individual predicted risk of developing sight-threatening DR. Its implementation in a Norwegian eye clinic over five years demonstrated safe and effective recommendation of variable screening intervals up to 23 months, compared to fixed 14-monthly screening intervals [29]. This type of individualized DR screening approach is very promising, and if properly validated, can be a major tool for resource optimization in the face of increasing DR disease burden.

#### AI models for diagnosis of DME

DME is the most common cause of visual impairment related to DR [30]. Timely diagnosis and accurate classification of DME are crucial to ensure appropriate treatment and to prevent further deterioration of vision. Current gold standard diagnostic technique for DME is optical coherence tomography (OCT). Recent works have leveraged DL methods for DME diagnosis from OCT scans [31, 32]. Tang et al. developed a multitask DL system using 73,746 OCT images from three commercially available OCT devices. This system demonstrated AUC values of 0.937–0.965 for DME detection, and 0.951–0.975 for center-involved DME (CI-DME) differentiation across these devices [33].

Although OCT is the definitive imaging standard for diagnosing DME, its widespread use as a screening tool is hampered by factors such as high cost and limited accessibility. In contrast, fundus photography is a more feasible option in primary care settings, owing to its widespread availability and cost-effectiveness. This provides a more accessible avenue for DME screening. Varadarajan et al. trained a DL model using 6039 fundus images from 4035 patients to predict CI-DME. This model delivered an AUC of 0.89, achieving 85% sensitivity at 80% specificity, surpassing even retinal specialist performance [34]. Additionally, for quantifying OCT metrics through fundus photos as input, DL models showcased high accuracy at specific macular thickness cut-offs [35], indicating the potential for quantifying severity of DME as well, which may be useful for triage in teleophthalmology contexts.

#### Automated identification and quantification of DME biomarkers

Previous clinical research has identified myriad useful DME biomarkers, including subretinal fluid (SRF), intraretinal fluid (IRF), pigment epithelial detachment (PED), hyperreflective dot (HRD), disorganization of inner retinal layers (DRIL), and disruptions in the external limiting membrane (ELM), ellipsoid zone (EZ), and cone outer segment tip (COST) [36–39]. Though the clinical utility of these biomarkers has been demonstrated in many studies, the detection and quantification of these biomarkers can be tedious or impractical in daily practice. Therefore, there is great interest in leveraging AI technologies for automated analysis of these biomarkers to inform disease prognosis and treatment decisions.

As an important feature for evaluating DME and determining treatment strategy, the retinal fluid become the primary area of interest for research. In contrast to earlier approaches, recent studies have adopted DL architectures for the segmentation and quantification of these fluid areas [40]. Furthermore, the automated quantification of the retinal fluid can also be used to predict visual acuity (VA) [41, 42]. These models offer significant improvements by reducing the need for subjective and labor-intensive manual annotations and providing more accurate segmentations. Unlike traditional methods that depend on low-level features sensitive to image quality, DL models learn to identify features at multiple levels automatically, marking a shift towards more efficient and unbiased research in DME.

Other biomarkers were also areas of interest [43–45]. Singh et al. implemented a DL method to detect DRIL, with an accuracy of 88.3% [45]. Orlando et al. developed a DL model to quantify the photoreceptor disruption on B-scans [43]. Multiple algorithms were developed using various techniques for automated detection of HRD in B-scans. However, their performances exhibit significant variability, with Dice similarity coefficient values ranging from 0.46 to 0.91 [46–48], which could be due to the relatively small size of the lesions. Further studies are required to enhance the precision of subtle lesion detection.

#### AI models for prediction of DME treatment response

The therapeutic approach of DME has evolved significantly over the past two decades. Intravitreal anti-vascular endothelial growth factor (anti-VEGF) agents have been established as the first-line treatment option for CI-DME with vision loss [49, 50]. However, there still exists significant heterogeneity in treatment response to anti-VEGF agents for individual patients caused by risk factors including morphological subtype, baseline VA, and concomitant treatments [51, 52]. This variability in treatment outcomes can pose a risk to adherence and ultimately, patient satisfaction.

Recent research has attempted to use AI for longitudinal predictions, focusing on treatment needs and analyzing both structural and functional outcomes. Cao et al. developed a model to predict the treatment response to three consecutive anti-VEGF injections in DME. The model utilized DL to autonomously extract OCT biomarkers, followed by the application of multiple classifiers for response prediction. The random forest model showcased superior results with a sensitivity of 0.900, a specificity of 0.851, and an AUC of 0.923 in predicting good responders and poor responders, even surpassing the predictive ability of ophthalmologists [53]. Alryalat et al. built a model composed of a modified U-net and an EfficientNet-B3 for a similar task, which achieved an accuracy of 75% for classification of treatment responders [54]. Moosavi et al. developed an automated software for analyzing vascular features in UWF fluorescein angiography (UWFFA) images to predict treatment outcomes in DME. They reported AUCs of 0.82 and 0.85 for morphological and tortuosity-based features, respectively, in discerning between treatment "rebounders" and "non-rebounders" [55]. Xie et al. used multiple datasets with OCT data combined with demographic and clinical data to predict 6-month post-treatment response and generate a recommendation to continue injection treatment. The algorithm achieved near-perfect structural prediction, and a mean absolute error (MAE) and mean squared error (MSE) of 0.3 to 0.4 logarithm of the minimum angle of resolution (logMAR) for visual outcome prediction. The accuracy of injection recommendations reached 70% [56]. Recently, Xu et al. used GANs to create post-treatment OCT images based on baseline images. The MAE of the central macular thickness comparing the synthetic and actual images was 24.51 ± 18.56 μm [57]. While the observed difference was not negligible, this study underscores the potential of GANs for structural predictions in ophthalmology. Such AI models, when integrated into clinical practice, could enable ophthalmologists to design more personalized treatment regimens tailored to each patient's unique retinal characteristics. This potentially improves not only the safety of therapy but also enhances the quality of life and creates potential cost savings for patients.

#### Prediction of visual function in DR and DME

Recent studies have been exploring the use of fundus images to assess visual function [58–60]. Kim et al. developed an ML-based VA measurement model using fundus photos from 79,798 patients with different retinal diseases, including DR. Images were divided into four VA categories, and the model demonstrated an average accuracy of 82.4% in estimating these four VA levels [58]. Recently, Paul et al. employed various AI architectures for predicting best-corrected visual acuity (BCVA) using fundus photos in CI-DME patients. The ResNet50 architecture showed the ability to estimate BCVA with an MAE of 9.66 letters, which is within two lines on the ETDRS chart. Additionally, the study observed that incorporating additional clinical visit data could potentially improve predictive accuracy, especially in the subset of patients with lower BCVA [59]. This approach could offer VA estimation for patients unable to participate in chart-based assessments. Moreover, estimating visual function with fundus photos in DR and DME appears promising, given the critical role of BCVA in guiding treatment decisions. However, the field is currently limited by sparse research, raising questions about the generalizability of findings. Consequently, further investigation is essential to validate and expand our understanding in this area.

### Telemedicine and remote monitoring

Telemedicine utilizes digital technology to provide healthcare services from distance, allowing patients to access medical consultations and treatments without visiting a healthcare facility. This method greatly improves medical care accessibility, particularly in underserved and rural regions, by overcoming geographic barriers between patients and providers [61]. The integration of AI-based DR screening systems further amplifies telemedicine's potential, offering more effective and efficient diagnostic capabilities. The current applications of screening for DR and DME in the primary care setting are based largely on the analysis of large data throughput collected via conventional retinal fundus or UWF cameras [62]. These approaches are potentially limited in scalability by resource issues such as high cost or shortage of trained personnel either to acquire images or to grade them, especially in remote areas.

The development of hand-held cameras and smartphone-based cameras opened up greater means of accessibility for fundus screening, by overcoming geographical barriers and providing patients in remote or underserved areas access to specialized care [63]. However, the potential for decreased image resolution and quality can be a concern [64].

Efforts have been undertaken to employ hand-held camera-based and smartphone-based fundus images in the field of telemedicine. In a comparative analysis, Ruan et al. evaluated the proficiency of a DL algorithm in identifying RDR from 50° fundus images taken via hand-held cameras against those obtained from traditional desktop cameras. While hand-held devices produced images of superior clarity that were effectively interpreted by human graders, the AI analysis revealed a need for further refinement [65]. Similarly, Rogers et al. reported reduced accuracy in DR detection using hand-held camera images with the Pegasus AI system (Visulytix Ltd., UK) [66]. In contrast, the SMART India Study Group described a DL model in detecting RDR using 2-field fundus photos acquired by nonmydriatic hand-held cameras from 16,247 eyes. The system achieved a high performance in detecting RDR, with an AUC of 0.98 and 0.99 with one-field and two-field inputs, respectively [67]. Significantly, they found that variations in dilation states and image gradability across studies could influence the results [65, 67, 68]. In a recent study, the SELENA + algorithm (EyRIS Pte Ltd, Singapore), which was developed using traditional fundus photos, was integrated into a hand-held fundus camera and paralleled the results of conventional retina specialist evaluations, reinforcing its precision in DR detection in a different use setting [69]. As for smartphone-based images, a study utilizing a DL algorithm previously trained on 92,364 traditional fundus images was subsequently run on 103 smartphone-captured images of varying qualities at 1080p resolution. It found that the algorithm achieved 89.0% sensitivity (95% CI: 81%–100%) and 83% specificity (95% CI: 77%–89%) for the detection of RDR. This was in spite of the presence of multiple glares, smudge and blockage artifacts that at times even required cropping of the retinal images prior to analysis [70]. Sosale et al. evaluated an offline DL-based DR screening software Medios (Medios technology, Singapore) in a clinical setting in India, which demonstrated promising results. This highlights its significant potential for deployment in areas with limited internet resources [71]. These results are encouraging, but more studies, especially based in real-world settings are required to further test this proof of concept. However, if robustly validated, they could be an important tool for increasing access to DR screening in under-resourced healthcare settings, as a means of convenience and cost-effectiveness.

In addition, home-based imaging devices serve as powerful tools for remote monitoring. These devices offer increased accessibility, especially for patients in remote areas, and provide the convenience of conducting regular scans from home, which is particularly beneficial for elderly or mobility-impaired patients. Home-based monitoring facilitates early detection and intervention, potentially preventing disease progression. Furthermore, it contributes to reduced healthcare costs by minimizing frequent clinic visits and integrates seamlessly with telemedicine, enhancing patient engagement and compliance. A home OCT system, Notal Vision Home OCT (NVHO, Notal Vision Inc, Manassas, VA, USA), was validated for daily self-imaging in patients with neovascular age-related macular degeneration [72]. Fifteen participants who were under anti-VEGF treatment performed daily self-imaging at home using NVHO for three months. Images were uploaded to the cloud and analyzed by its DL-based analyzer. Results indicated good agreement between the analyzer and human experts on fluid status in 83% of scans, and 96% agreement between the home-based OCT and in-clinic OCT scans. While similar results have been reported in clinical settings for DME [73], further studies are needed to validate the system's efficiency in a home-based setting.

### Technical AI advancements and innovations for DR and DME

In the realm of ophthalmology, technical advancements and innovations in AI are paving the way for groundbreaking improvements. These technological breakthroughs are enhancing accuracy and efficiency in the detection and clinical evaluation of these diabetes-related eye conditions, and providing a more explicit understanding of how AI works for DR and DME management.

A notable development in this area is the application of generative AI, which represents a significant advancement in the field. Generative AI, including techniques like naïve Bayes and linear discriminant analysis, has seen renewed interest, particularly with applications like ChatGPT and in the medical imaging domain through diffusion models and GANs, for tasks including classification, segmentation, and image synthesis [74].

**Fig. 2:**
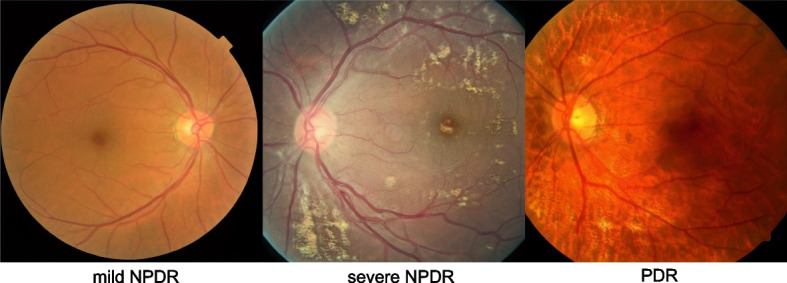
Examples of synthetic fundus photography of diabetic retinopathy generated by generative adversarial network

For DR and DME, a common methodology has been to train a GAN with existing retinal fundus images. The trained GAN can then be used to generate synthetic retinal fundus images from the learnt distribution. These synthetic images can then be considered as additional unique image data, with various possible uses. First, these images serve as data augmentation, particularly valuable for addressing imbalances in dataset distributions across DR severity classes. Figure 2 displays a variety of synthesized fundus photographs, each representing different classes of DR. Zhou et al. introduced a GAN with multi-scale spatial and channel attention module, to allow arbitrary grading and lesion label manipulation in the GAN latent space [75]. The synthetic images were then employed to successfully improve the performance of pixel-level segmentation models. Lim et al. formulated a MixGAN model that attempts to automatically adjust the synthetic data distribution to optimize classifier performance [76]. Balasubramanian et al. augmented the rare PDR class in their dataset with additional synthetic images generated by a deep convolutional GAN (DCGAN) model, towards improving classification performance [77]. Synthetic images can function as a training dataset, enabling not just the achievement of balanced data for improved training, but also the replacement of real data to safeguard patient privacy.

Second, GANs may be used to generate internal features, variations or masks of the input image that are then indirectly used to aid discrimination. Zhang et al. formulated an encoder-decoder module within a GAN to generate normal lesion-free versions of potential-DR input images, and compared the generated "fake normal" image against the input image with a classifier model [78]. Wang et al. proposed a multichannel semisupervised GAN (SSGAN), which combines multiple parallel generators that produce a series of subfundus images, which include effective DR features [79]. These generated DR features are then compared against actual features extracted from the input fundus image, with a discriminator model. Xiao et al. incorporated HEDNet as a generator into a conditional PatchGAN-based model, to help refine lesion segmentation results [14].

Third, synthetic images generated by GANs may be used to aid the training of human graders in diagnosing DR and DME, especially where specific combinations of lesions or underlying factors that are rarely found in reality, are required. In such cases, the realism of the synthetic images becomes important. Chen et al. evaluated the ability of human experts to distinguish retinal images generated by a pix2pixHD model using retinal vessel maps from real retinal images [80]. They found that only 59% of the images were correctly identified, suggesting that in most cases, human experts could not reliably discern real from synthetic images. Tavakkoli et al. demonstrated translation between different image modalities for DR with a conditional GAN in producing FFA images from fundus photographs [81]. Such domain transfer is potentially useful for generating more information from existing imaging modalities.

A continuing concern accompanying the use of AI models for DR and DME detection and screening in practice has been that model decisions are generally not directly understandable and auditable by humans. This has implications in establishing the robustness of AI models with data from different sources, and also culpability in the case of incorrect predictions. As such, explainable AI (XAI) has understandably become an important consideration in AI model acceptance [82].

XAI techniques are commonly categorized as either model-agnostic or model-specific, model-based or post-hoc, and global or local [83]. Model-specific approaches depend on a particular AI model, whereas model-agnostic methods apply across AI models by examining input–output relationships, effectively viewing the model as a "black box". Model-based strategies are inherently interpretable due to simpler features or logic, while post-hoc methods impose abstractions for explanation. Global techniques offer overarching insights learned by the AI, whereas local ones explain individual inputs. Since modern DR and DME AI models tend to involve image inputs to deep neural network architectures, saliency heatmap techniques such as Grad-CAM [83] and Integrated Gradients [84] – which are model-specific, post-hoc, local techniques – have tended to be the explainability method of choice [76].

Challenges for XAI in DR and DME stem from the small scale of lesions, like microaneurysms, against the larger retinal image, making it difficult for techniques like Grad-CAM to precisely highlight these features. To address this, research has shifted towards learning lesion segmentation directly [85]. Approaches include integrating VGG16 encoders with U-Net for joint DR classification and lesion segmentation, employing modified U-Nets for semantic segmentation, and using transformers to capture small lesion patterns [86–89]. However, a drawback of segmentation as an XAI approach is that it requires annotation of individual lesions or lesion regions, which is not usually performed in DR grading, except in certain research datasets. To avoid this requirement, Quellec et al. introduced the ExplAIn algorithm, an end-to-end model that learns the separation between foreground/lesions and background pixels through self-supervision [90]. This requires only image-level labels, but allows pixel-level segmentations to be associated with the image-level labels through simple rules.

Integrating XAI into DR and DME algorithms offers several clinical advantages, including increased trust and adoption among clinicians, improved decision-making through deeper insights into diagnostic and prognostic factors, and easier model validation. XAI also supports regulatory compliance by providing transparency and accountability in AI-driven decisions, allows for personalized patient care through tailored treatment plans, and serves as an educational tool that enhances clinician understanding of disease patterns. Overall, XAI has the potential to bridge the gap between complex AI algorithms and clinical applications, facilitating more informed, ethical, and patient-centric healthcare practices.

### Current challenges and future direction

While the volume of solid evidence supporting the efficacy of AI tools in diagnosing and monitoring these conditions is growing, a substantial gap remains in translating these technological advances into routine clinical practice (Table 1). The discrepancy between research findings and real-world performance, along with variations in effectiveness across different ethnicities and regions, underscores a critical issue [6]. Ethical and privacy concerns also play a pivotal role in the slow adoption of AI in clinical practice. With AI's reliance on vast datasets for training algorithms, as well as implementation with clinical data, ensuring the privacy and security of patient data is critical [91]. Furthermore, the 'black box' nature of some AI algorithms, which do not readily reveal how decisions are made, raises ethical questions about transparency and accountability in patient care decisions [92].

**Table 1:** Current challenges and future direction for research in diabetic retinal diseases

Current Challenges	Future direction
Intrinsic performance limitation	Expand research on enhancing the core intelligence of AI models to ensure they maintain accuracy and reliability when exposed to a broad range of clinical data
Regulatory barriers	Collaboration between AI developers, healthcare professionals and regulatory agencies to create a comprehensive framework for the evaluation and certification of AI-based medical devices and systems
Ethical and privacy concerns	Advance the use of anonymization and synthetic data techniques Establish clear guidelines and protocols for data handling that build public trust
Implementation difficulties	Implementation with proper strategy to ensure cost-effectiveness Create educational programs and interactive platforms that facilitate clinician engagement

Another challenge is navigating the regulatory landscape. AI applications in healthcare should undergo rigorous regulatory review, ensuring they adhere to the highest standards of safety and efficacy [93]. For AI tools targeting DR and DME, this means undergoing rigorous clinical trials and validation studies to satisfy the requirements of regulatory bodies like the Food and Drug Administration (FDA). However, the dynamic nature of AI and the uncertainty in defining the responsibility of AI poses unique challenges for regulatory approval.

Moreover, the integration of AI into clinical settings also faces practical challenges, such as healthcare staff's bias against new AI methods, and uncertainty of how the AI tools should be integrated into clinical work for healthcare professionals unfamiliar with AI [94, 95]. Addressing these issues requires comprehensive training programs and initiatives to demonstrate the tangible benefits of AI in enhancing patient care. Additionally, the varied applications of AI tools in clinical settings can influence their cost-effectiveness, presenting another obstacle to their real-world implementation [96, 97].

To bridge the gap between evidence and clinical implementation, a comprehensive approach is needed. This includes promoting closer collaboration between AI developers, regulatory bodies, and healthcare professionals to streamline the approval process and ensure AI tools meet clinical needs. Developing clear guidelines and standards for AI in healthcare, alongside robust training programs for healthcare providers, can also accelerate adoption. Additionally, addressing ethical and privacy concerns through transparent AI algorithms, stringent data protection measures, and promoting the use of generative training data will be crucial in gaining public trust and acceptance.

## Conclusion

The development of novel AI algorithms has tremendous transformative potential in the management of DR and DME. These state-of-the-art algorithms have the potential to enhance accuracy in screening, triage, and diagnosis and also allow for AI-driven precision medicine by enabling longitudinal prediction of disease progression and recommending tailored interventions. As DR screening systems advance and are progressively implemented in real-world settings, transition of burden from primary care to specialized medical facilities is expected. Consequently, there is a pressing need for tertiary care clinicians to arm themselves with higher efficiency AI tools. Moving forward, future DR and DME AI research endeavors should focus on a unified and thorough regulatory framework that ensures effectiveness and safety in a broad range of real-world contexts through proper deployment methods. This involves refining patient evaluations, offering more precise prognostic predictions, improving risk stratification, and providing personalized recommendations for follow-up care and treatment. Embracing AI-based precision medicine is essential in addressing the escalating global burden of DR and DME.

## Data Availability

Not applicable.

## Abbreviations

DR: Diabetic retinopathy
DME: Diabetic macular edema
AI: Artificial intelligence
DM: Diabetes mellitus
FDA: Food and Drug Administration
RDR: Referable diabetic retinopathy
UWF: Ultra-widefield
ETDRS: Early Treatment of Diabetic Retinopathy Study
DL: Deep learning
AUC: Area under the receiver operating characteristic curve
ML: Machine learning
VA: Visual acuity
CI-DME: Center-involved diabetic macular edema
BCVA: Best-corrected visual acuity
MAE: Mean absolute error
OCT: Optical coherence tomography
CNN: Convolutional neural network
HRD: Hyperreflective dot
DRIL: Disorganization of inner retinal layers
ELM: External limiting membrane
EZ: Ellipsoid zone
COST: Cone outer segment tip
VEGF: Vascular endothelial growth factor
MSE: Mean squared error
logMAR: Logarithm of the minimum angle of resolution
GAN: Generative adversarial networks
UWFFA: Ultra-widefield fluorescein angiography
OCTA: Optical coherence tomography angiography
SSGAN: Semisupervised generative adversarial networks
XAI: Explainable artificial intelligence
